# Systematic review of patient factors affecting adipose stem cell viability and function: implications for regenerative therapy

**DOI:** 10.1186/s13287-017-0483-8

**Published:** 2017-02-28

**Authors:** Jajini Varghese, Michelle Griffin, Afshin Mosahebi, Peter Butler

**Affiliations:** 1 Charles Wolfson Center for Reconstructive Surgery, Royal Free Hospital, London, UK; 2 UCL Centre for Nanotechnology and Regenerative Medicine, Division of Surgery & Interventional Science, University College London, London, UK

**Keywords:** Adipose-derived stem cells, Body mass index, Age, Stem cell therapy, Stem cell viability

## Abstract

**Background:**

The applications for fat grafting have increased recently, within both regenerative and reconstructive surgery. Although fat harvesting, processing and injection techniques have been extensively studied and standardised, this has not had a big impact on the variability of outcome following fat grafting. This suggests a possible larger role of patient characteristics on adipocyte and adipose-derived stem cell (ADSC) viability and function. This systematic review aims to collate current evidence on the effect of patient factors on adipocyte and ADSC behaviour.

**Methods:**

A systematic literature review was performed using MEDLINE, Cochrane Library and EMBASE. It includes outcomes observed in in vitro analyses, in vivo animal studies and clinical studies. Data from basic science work have been included in the discussion to enhance our understanding of the mechanism behind ADSC behaviour.

**Results:**

A total of 41 papers were included in this review. Accumulating evidence indicates decreased proliferation and differentiation potential of ADSCs with increasing age, body mass index, diabetes mellitus and exposure to radiotherapy and Tamoxifen, although this was not uniformly seen across all studies. Gender, donor site preference, HIV status and chemotherapy did not show a significant influence on fat retention. Circulating oestrogen levels have been shown to support both adipocyte function and graft viability. Evidence so far suggests no significant impact of total cholesterol, hypertension, renal disease, physical exercise and peripheral vascular disease on ADSC yield.

**Conclusions:**

A more uniform comparison of all factors highlighted in this review, with the application of a combination of tests for each outcome measure, is essential to fully understand factors that affect adipocyte and ADSC viability, as well as functionality. As these patient factors interact, future studies looking at adipocyte viability need to take them into consideration for conclusions to be meaningful. This would provide crucial information for surgeons when deciding appropriate volumes of lipoaspirate to inject, improve patient selection, and counsel patient expectations with regards to outcomes and likelihood for repeat procedures. An improved understanding will also assist in identification of patient groups that would benefit from graft enrichment and cryopreservation techniques.

**Electronic supplementary material:**

The online version of this article (doi:10.1186/s13287-017-0483-8) contains supplementary material, which is available to authorized users.

## Background

Autologous fat grafting has become a standard treatment for volume and contour defects in reconstructive surgery [1]. In 1983, Illouz and Sterodimas first described fat grafting, where the graft was obtained from a donor site through liposuction and re-injected immediately into the region of interest through syringes [2, 3]. Stem cells isolated from bone marrow (BM-MSCs) are the most characterised and clinically studied stem cell source to date [4]. Passage number, expansion medium, culture conditions and stem cell source all influence mesenchymal stem cell (MSC) characteristics [5–7].

The painful isolation and low yield associated with BM-MSCs has prompted research into other stem cell sources. In 2001, Zuk et al. [8] isolated MSCs from adipose tissue with the same potential as BM-MSCs to differentiate not only into mesenchymal lineages, such as adipogenic, chondrogenic [9, 10], osteogenic [11, 12], myogenic [13] and cardiomyogenic [14] lines, but also into neurogenic [15], angiogenic [16, 17] and hepatic lineages [18]. Adipose-derived stem cells (ADSCs) also display immunosuppressive, anti-inflammatory and angiogenic properties through the release of soluble mediators in a paracrine fashion [19]. This together with the ease of isolation and abundant supply makes ADSCs attractive not only in the regenerative field but also as a tool to enhance the survival of fat grafts [20]. However, fat grafting has two main limitations, inconsistency with fat graft survival and poor reliability [21, 22].

Although fat harvesting, processing and injection techniques have been extensively studied and standardised, this has not had a big impact on the variability of outcome following fat grafting [23]. This suggests a possible larger role of patient characteristics on ADSC number, viability and functionality. This systematic review aims to collate evidence on the effect of patient factors on adipocyte and ADSC viability and functionality.

The isolation procedure for adipose tissue results in a stromal vascular fraction (SVF) layer that is composed of a host of cells, including stem cells, pericytes, monocytes, macrophages and capillary endothelial cells. Dominici et al. [24] provided the criteria to identify MSCs, which include plastic adherence, the expression of CD105, CD73 and CD90, a lack of expression of CD45, CD34, CD14 or CD11b, CD79alpha or CD19 and HLA-DR surface molecules and the ability to differentiate into osteoblasts, adipocytes and chondroblasts in vitro.

To avoid confusion through terminology, we refer to multipotent precursor cells from adipose tissue stroma as adipose-derived stem cells (ADSCs) [25].

## Methods

**Fig. 1:**
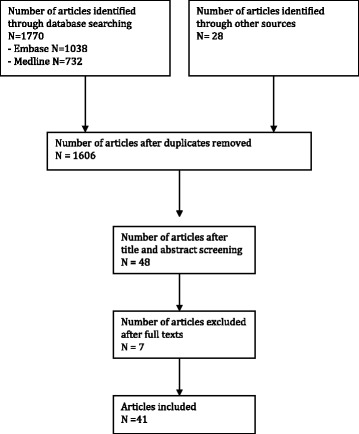
Flow chart to demonstrate paper selection in this study

An electronic search of the MEDLINE through PubMed and EMBASE databases was performed to identity all original clinical papers from 1959 to 2016 that described effects of patient factors, medication or systemic conditions on adipocyte viability, proliferation and differentiation potential (reviewed by two independent reviewers, J.V. and M.G.). For the same time period, all in vitro studies that assessed the effect of patient factors on adipocyte and ADSC function were identified. Keywords with Boolean operators used in the search included the following: “adipocyte” or “stem” or “ADSC” or “lipoaspirate” and “age” or “BMI” or “radiotherapy” or “diabetes” or “menopausal status” or “donor sites” or “HIV” or “cardiovascular disease” or “renal disease” or “gender”. The full search strategy is provided in Additional file 1: Table S1 and Fig 1.

Articles were considered eligible if they met the following inclusion criteria: (1) clinical, in vitro and animal studies that evaluated the effect of patient factors on adipocyte or ADSC yield and or function; (2) the outcome measures included adipocyte or ADSC or SVF yield and/or function (differentiation and proliferation capacity).

Articles were excluded if they were: (1) papers describing effects of patient factors on stem cells of other origin, such as BM-MSCs; (2) studies not published in English, as the reviewers could not fully understand the manuscript; (3) editorials, publications on congress meetings, unpublished data or letters to the editor. Review articles were only used to contribute to the “Discussion” section and identify any other relevant articles.

A variety of names were used in the included studies to describe the plastic-adherent cell population isolated from collagenase digests of adipose tissue. These included adipose-derived stem/stromal cells (ASCs), adipose-derived adult stem (ADAS) cells, adipose-derived stromal cells (ADSCs), adipose stromal cells (ASCs), adipose mesenchymal stem cells (AdMSCs), lipoblasts, pericytes, preadipocytes and processed lipoaspirate (PLA) cells. To avoid confusion we decided to adopt the term “adipose-derived stem cells” (ADSCs) to identify the isolated, plastic-adherent, multipotent cell population as recommended by the International Fat Applied Technology Society.

Studies were assigned a Level of Evidence (LOE) adapted from the Oxford Centre for Evidence Based Medicine (http://www.cebm.net/index.aspx?o=1025) to establish whether valid and reliable evidence is present for effects of patient factors on adipocyte viability. These levels, ranging from LOE-1 to LOE-5, are based on methodology and study design. In brief, LOEs were assigned as follows: LOE 1 = randomized control trial; LOE-2 = cohort study or cross-sectional study in consecutive participants; LOE-3 = case–control study; LOE-4 = case series study; LOE-5 = animal studies, expert opinion or case report [26].

## Results

The search for patient factors that affect SVF, adipocyte or ADSC yield and/or function led to the identification of the following factors described in the following sections: age, body mass index (BMI), gender, menopausal status, donor sites, HIV status and cancer treatments, including radiotherapy, chemotherapy and tamoxifen usage.

### Age

Sixteen LOE-2 in vitro studies of human ADSCs and three LOE-5 animal studies reported on the effect of age on ADSC viability and function (Table 1). Twelve of these studies did not identify any effect on adipocyte yield [27–36]. However, more recent studies using gene expression measurements of senescence have shown a significant decrease in overall yield of nucleated cells with increasing age [37, 38] and, more potently, a significant decrease in the proliferative and differentiation capacities of ADSCs [35, 38–41]. Madonna et al. [42] compared omental ADSCs between ‘young’ (*n* = 18, 40–54 years) and ‘elderly’ (*n* = 22, 66–92 years) and reported significant decreases in ADSC yield and angiogenic capacity with increasing age. While Zhu et al. [43] did not find a significant effect on the adipogenic potential of ADSCs, advancing age significantly reduced osteogenic potential. This is supported by other studies that have reported similar age-dependant decreases in the osteogenic potential of ADSCs [9, 44]. Such age-dependency of differentiation capacity has also been observed in rat ADSCs [45–47].

**Table 1:** Studies that have evaluated the association between age and human ADSC and adipocyte functionality (ordered by sample size)

Reference	LOE	Sample size (*n*)	Subjects	F (*n* or %)	Age (years; mean ± SD or only mean)	Age (years) (subset categories)	Outcome: ADSC yield	Outcome: differentiation potential
van Harmelan et al. 2003 [34]	2	189	Elective breast reduction procedure	100%	16–73	-	No significant correlation between age and ADSCs per gram of tissue	No significant correlation between age and ADSC differentiation
Faustini et al. 2010 [29]	2	125	Donor sites varied by gender. Men had more resected samples; women had more lipoaspirates	70%	15–87 (51.31)	-	No significant correlation between age and ADSC yield (stratified by gender)	-
Yu et al. 2010 [36]	2	64	Elective liposuction surgery or abdominoplasty	90.6%	18–66 (43.6 ± 11.1)	-	No significant correlation between age and ADSC yield	-
Madonna et al. 2011 [42]	2	52	Patients undergoing abdominal surgery with varying cardiovascular history	38%	68 ± 13	40–54 (12) 55–65 (18) 66–92 (22)	Significant negative correlation between age and ADSC yield (subset matched for co-morbidities)	
Harris et al. 2010 [30]	2	50	Lipoaspirate from abdomen in patients undergoing elective vascular procedures Multiple co-morbidities	36%	59 ± 16	<70 (35) >70 (15)	No significant correlation between age and ADSC yield	No significant correlation between ADSC differentiation and age
Alt et al. 2012 [38]	2	40	Adipose tissue from healthy donor tissue	-	15–71	<20 (15) 30–40 (17) > 50 (8)	Significant negative correlation between age and ADSC yield	Significant negative correlation between age and ADSC proliferation and differentiation rate
Mojallal et al. 2011 [31]	2	42	Elective liposuction—abdomen	100%	27–71	-	No significant correlation between age and ADSC yield and proliferation	-
Choudhery et al. 2014 [37]	2	29	Lipoaspirate from healthy men and women	69%	24–67	<30 (8) 35–50 (10) > 60 (11)	Significant negative correlation between donor age and ADSC yield	Significant negative correlation between age and ADSC proliferation rate Adipogenic differentiation was independent of age Osteogenic and chondrogenic potential decreased with age
Hauner et al. 1989 [121]	2	27	Subcutaneous tissue from patients undergoing elective abdominal surgery	37%	20–83	-	Significant negative correlation between age and ADSC yield (in both genders)	Significant negative correlation between age and ADSC proliferation (in both genders)
de Girolamo et al. 2009 [122]	2	26	Lipoaspirate from healthy women	100%	21–68	<35 (12) > 45 (14)	Significant positive correlation between age and ADSC yield	Proliferation rates higher in younger women but not statistically significant No significant impact of age on adipogenic differentiation potential Significant negative correlation of age with osteogenic potential
Padoin et al. 2008 [32]	2	25	Elective liposuction—multiple donor sites	100%	21–37 (30.7 ± 4.3)	-	No significant correlation between age and ADSC yield	-
Geissler et al. 2014 [27]	2	24	Lipoaspirate from healthy women. Multiple donor sites	100%	25–71 (51)	≤45 (9) ≥ 46 (15)	Analyses stratified by donor site: Abdomen: younger women showed significantly higher yield Flanks: older women had significantly higher ADSC yield	-
Aust et al. 2004 [28]	2	18	Elective liposuction	87.5%	20.5–29.3 (24.9 ± 2.7)	-	No significant correlation between age and ADSC yield	-
Schipper et al. 2008 [33]	2	12	Lipoaspirate from body-contouring surgery in previously obese patients	100%	25–60	25–30 (3) 40–45 (4) 55–60 (5)		Higher proliferation of ADSC and lower susceptibility to apoptosis in 20 year old group (not statistically significant)
Yoshimura et al. 2006 [35]	2	-	-	100%	21–59	-	No significant correlation between ADSC yield and age	-
Zhu et al. 2009 [43]	2	-	Lipoaspirate from multiple donor sites	100%	20–58	-	-	Slower rate of proliferation with increasing age (not statistically significant) Significant decrease in osteogenic potential

*ADSC* adipocyte-derived stem cell, *F* female, *LOE* Level of Evidence, *n* number, *SD* standard deviation

### Body mass index

Fourteen LOE-2 studies investigated the effect of BMI on adipocyte viability. Eight studies demonstrated an effect of increasing BMI on adipocyte viability and function [28, 34, 48–52] (Table 2). In the largest study to date (*n* = 189), with 30 women within the ‘obese’ category (BMI >30 kg/m^2^), van Harmelan et al. [34] reported a significant reduction in the number of viable mature adipocytes per gram of adipose tissue and in the differentiation capacity of ADSCs with increasing BMI. This finding is supported by five other in vitro studies that also showed decreases in both differentiation and proliferation capacities of adipocytes with increasing BMI [28, 34, 48, 49, 51] (Table 2). Frazier et al. reported ADSCs from obese individuals were compromised in early adipogenic and osteogenic potential and correlated this with their potential to form colonies in vitro, which was inversely proportional to the individual’s BMI.

**Table 2:** Studies that have evaluated the association between BMI and human ADSC and adipocyte functionality (ordered by sample size)

Reference	LOE	Sample size (*n*)	Subjects	Female (*n* or %)	BMI (kg/m^2^)	BMI: subset categories (*n*)	Outcome: ADSC yield	Outcome: differentiation potential
Van Harmelan et al. 2003 [34]	2	189	Healthy women undergoing breast reduction procedure	100%	19.7–39.7	<25 kg/m^2^ (57) 25–29.9 kg/m^2^ (96) > 30 kg/m^2^ (35)	Significant positive association between BMI and ADSC count per body Significant negative correlation between BMI and ADSC per gram of adipose tissue	Significant negative correlation between BMI and ADSC differentiation potential
Faustini et al. 2010 [29]	2	125	Men had more resected samples; women had more lipoaspirates. Donor sites also varied with gender. Therefore, analyses were stratified for gender	88	25.33 ± 3.44 (M) 26.68 ± 5.4 (F)	-	No significant correlation between ADSC yield and BMI in both males and females	-
Yu et al. 2010 [36]	2	64	Elective liposuction surgery or abdominoplasty	90.6%	18.3–37.2 (27.0 ± 3.8)	<25 kg/m^2^ (6) 25–29.9 kg/m^2^ (6) > 30 kg/m^2^ (6)	Donor BMI was associated with increased ADSC yield per unit volume of lipoaspirate tissue	No significant difference in ADSC proliferation in subset analyses (n = 6 each cohort)
Harris et al. 2010 [30]	2	50	Lipoaspirate from abdomen in patients undergoing elective vascular procedures. Multiple co-morbidities	36%		<30 kg/m^2^ (30) ≥ 30 kg/m^2^ (20)	No significant correlation between ADSC yield and BMI	-
Isakson et al. 2009 [49]	2	51	Abdominal subcutaneous tissue: needle biopsy (45) and bariatric surgery (6)	-	19.3–54.8 (28.8 ± 2.2)	-	-	Significant negative correlation between BMI and adipogenic differentiation potential
Mojallal et al. 2011 [31]	2	42	Elective liposuction—abdomen	100%	20–35.6 (26.3)	≤25 kg/m^2^ (15) > 25 kg/m^2^ (27)	No significant correlation between BMI and ADSC yield	Tendency toward negative correlation between BMI and proliferation rate (not statistically significant)
Padoin et al. 2008 [32]	2	25	Elective liposuction—multiple donor sites	100%	20–37 (26.2 ± 4.4)	-	No significant correlation between BMI and ADSC yield (adjusted for donor site)	-
Geissler et al. 2014 [27]	2	24	Elective liposuction—multiple donor sites	100%	20.4–34.5	<25 kg/m2 (13) ≥ 25 kg/m2 (11)	No significant correlation between BMI and ADSC yield (adjusted for donor site)	-
Aust et al. 2004 [28]	2	18	Elective liposuction—hips and thighs	87.5%	20.5–29.3 (24.9 ± 2.7)	-	Significant negative correlation between BMI and ADSC yield	-
Roldan et al. 2011 [52]	2	16	Omental adipose tissue: 12 obese patients undergoing bariatric study and 4 lean patients undergoing abdominal surgery	50%	-	≤25 kg/m^2^ (4) 40–55 (6) ≥ 55 (6)	-	Significant negative correlation between BMI and ADSC proliferation Positive correlation between BMI, premature senescence and cytokine secretion
Frazier et al. 2013 [48]	2	12	Cryopreserved ASCs isolated from lipoaspirate from abdomen	100%	22.2 ± 1.79	≤25 kg/m^2^ (6) > 25 kg/m^2^ (6)	-	Significant negative correlation between BMI ADSC proliferation and osteogenic differentiation potential
Perez et al. 2013 [50]	2	10	Adipose tissue from patients after bariatric surgery	100%	20.0 ± 2.1 (<25 kg/m^2^) 34.0 ± 3.1(>30 kg/m^2^)	<25 kg/m^2^ (5) > 30 kg/m^2^ (5)	-	Significant negative correlation between BMI and ADSC differentiation and migration capabilities
Perez et al. 2015 [51]	2	10	Adipose tissue from patients after bariatric surgery	100%	-	<22 kg/m^2^ (5) > 30 kg/m^2^ (5)	Significant negative correlation between BMI and ADSC yield	Significant negative correlation between BMI and ADSC proliferation Significant changes seen in telomerase activity and DNA telomere length leading to reduced self-renewal capacity
Yoshimura et al. 2006 [35]	2	-		100%	-		No significant correlation between ADSC yield and BMI	-

*ADSC* adipocyte derived stem cell, *BMI* body mass index, *F* female, *LOE* Level of Evidence, *n* number

In addition to the reduced capacity for differentiation and migration and angiogenic and proliferative abilities of ADSCs from obese humans [50, 52, 53], Perez et al. [51] also noted changes in telomerase activity and DNA telomere length, suggesting a decreased self-renewal capacity and early apoptosis. Isakson et al. [49] and Tang et al. [54] showed that this reduction in differentiation of enlarged ADSCs may be related to increased mitogen-activated protein 4 kinase 4 (MAP4K4) expression, which inhibits peroxisome proliferator–activated receptor (PPAR)-γ activation and thereby adipogenesis.

It has been reported that after massive weight loss, subcutaneous adipose tissue returns to a non-inflammatory state with a significant decrease in cytokines [55]. Mitterberger compared ADSCs from ‘formerly obese’ patients who had undergone bariatric procedures to ‘obese’ and ‘normal weight’ individuals. They showed that bariatric surgery and diet-induced long-term calorie restriction substantially reprogrammed ADSCs, with reduced DNA damage, improved viability and extended replicative lifespan [56]. ADSCs isolated from ex-obese patients attained a mature adipocyte phenotype faster than those obtained from non-obese patients [57], suggesting an enrichment of cells in the ADSC population that are ‘more prepared’ for adipogenic differentiation.

Interestingly, six studies (LOE-2) reported no significant association with increasing BMI [27, 29, 31, 32, 35, 36]. Mojallal et al. reviewed in a prospective study 42 women with varying BMI. After dividing the patients into two groups (BMI ≤25 or >25 kg/m^2^), they did not find a statistically significant correlation between BMI and proliferation [31]. Similarly, Faustini et al. [29] analysed data from 125 subjects stratified by gender and did not see an association among increasing donor BMI and ADSC yield or function (Table 2).

### Gender

Ogawa et al. [58] showed in a LOE-5 study that PPAR-[gamma]2 expression levels (marker of adipogenesis) were 2.89 times greater in ADSCs harvested from female mice compared to male mice, raising the possibility of a significant role of gender. In vitro LOE-2 studies of human ADSCs, however, have so far not shown any difference in ADSC yield and proliferation by gender [29, 30, 59]. Faustini et al. [29] studied 37 males and 88 females and reported that the best donor site among men in terms of yield was the abdomen. Aksu et al. [60] studied abdominoplasty tissue from three males and three females and reported that ADSCs from males showed more effective osteogenic differentiation compared to those from females.

### Menopausal status

Three LOE-2 in vitro and one LOE-5 in vivo study in mice investigated the effect of menopausal status or oestrogen on adipocyte viability. Geissler et al. [27] reported increased adipocyte viability using lower abdominal fat from younger, presumably pre-menopausal women (<45 years) compared to from older women, suggesting a modulatory role of circulating oestrogen levels. However, information regarding hormonal status or supplements was not gathered.

To further examine the effect of circulating oestrogens on fat graft outcomes, the same group later harvested adipose tissue from inguinal pads of mice that underwent either ‘sham’ or ‘ovariectomy’ operations, which was then injected into another set of mice [61]. The fat grafts from mice that had the ‘sham’ procedure, and therefore circulating oestrogen, were softer and showed higher capillary density and higher expression of proangiogenic factors.

Interestingly, transfer of lipoaspirate into ‘sham’ or ‘ovariectomy’ recipients did not alter the weight or vascular density 45 days after transplantation, suggesting a smaller role of circulating oestrogen post-fat transfer [61]. Addition of 17β-oestradiol to ADSCs has been shown to significantly improve adipogenic differentiation with enhanced survival of fat transfer by reducing apoptosis in nude mice [62, 63].

### Donor site

The search for the ideal donor site for fat harvest is ongoing. So far ten LOE-2 in vitro studies using human ADSCs and one LOE-5 animal study have investigated donor site as a potential influence on adipocyte behaviour. Of the ten studies of human ADSCs, only three found any difference in adipocyte behaviour between different sites (Table 3). Padoin et al. [32] (*n* = 25) showed that fat from the lower abdomen and medial thighs has higher ADSC yield compared to the upper abdomen, trochanteric region, knees and flanks. Jurgens et al. [64] (*n* = 22) also reported significantly higher ADSC yield from abdominal aspirate with no significant differences in differentiation capacity. Geissler et al. [27] (*n* = 24) reported greater adipocyte viability in lipoaspirates from lower abdomen compared to from flanks and inner thighs, evident only in a subset of younger women (<45 years). There is some evidence to suggest higher ADSC yields from abdominal tissue compared to back and knee among men [29]. However, this difference was not seen among women. This is in agreement with previous studies suggesting that the choice of donor site has little effect on fat graft outcomes [32, 65–68]. Within the abdomen, fat superficial to the Scarpas layer displays increased multipotency and stemness features compared to a deep abdominal depot [33, 69].

**Table 3:** Studies that have evaluated the association between harvest site and human ADSCs and adipocyte functionality (ordered by sample size)

Reference	LOE	Sample size (*n*)	Subjects	Donor sites	Outcome: clinical	Outcome: ADSC yield	Outcome: differentiation potential
Small et al. 2014 [123]	2	73	Fat transfer to reconstructed breasts	Two donor sites: abdomen and thighs	Measured pre- and post-operative 3D scans to assess volumetric changes. No statistically significant difference between donor sites		
Lim et al. 2012 [124]	2	27	Patients with craniofacial microsomia, Treacher Collins syndrome	Two groups: abdomen and non-abdominal sites	Measured 2D analysis using pre-op and post-op photographs. Adipose tissue from abdominal or non-abdominal sources equally corrected asymmetry		
Padoin et al. 2008 [32]	2	25	Liposuction aspirate	Six donor sites: upper abdomen, lower abdomen, knee, inner thigh, flank and trochanteric region		The cell concentration in lower abdomen and inner thigh was statistically higher than in other areas	
Geissler et al. 2014 [27]	2	24	Liposuction aspirate	Three donor sites: lower abdomen, thigh and flank		In younger patients, adipocyte viability was greater in the lower abdomen than in the flank; in older patients, this difference was not seen. Higher viability of flank adipocytes in the older group compared with younger group	
Jurgens et al. 2008 [64]	2	22	Liposuction aspirate	Two donor sites: abdomen and hip/thigh		ADSC yield significantly higher from abdominal aspirate	No statistical difference in absolute number of nucleated cells and differentiation capacity
Rohrich et al. 2004 [67]	2	5	Liposuction aspirate	Three donor sites: knee, flank and abdomen		No statistical difference in viability	
Choudhery et al. 2014 [120]	2	3	Liposuction aspirate	Five donor sites: abdomen, flank, thigh, scarpas fascia, submental jowl		No statistical difference in proliferation	
Di Taranto et al. 2015 [125]	2	1	Liposuction aspirate	Superficial versus deep abdominal fat			Superficial fat increased differentiation capacity
Ullmann et al. 2005 [68]	2	45 nude mice (15 in 3 groups)	Fat from one woman injected into 3 groups with 15 nude mice each	Three donor sites: abdomen, breast and thigh		Fat compared for viability and volume retention showing no statistical difference	
Li et al. 2013 [66]	2	30 nude mice (6 in 5 groups)	Fat from six women injected into nude mice	Five donor sites: flank, upper abdomen, lower abdomen, medial thigh, lateral thigh viability	.	Cell count and assay by flow cytometry and graft evaluation at 12 weeks post-transplantation and showed no statistical difference	

*ADSC* adipocyte derived stem cell, *LOE* Level of Evidence, *n* number

### Radiotherapy, chemotherapy and tamoxifen

A LOE-5 study by Poglio et al. [70] investigated mice after whole body radiation and reported that adipose tissue can be deeply damaged by radiotherapy, significantly reducing both the number and proliferation capacity of ADSCs.

Administration of immunosuppressive medications such as anti-lymphocyte treatment and alemtuzumab (lytic monoclonal antibodies) and tacrolimus for lymphocyte depletion following composite tissue transplantation have been shown to decrease both the viability and proliferative capacity of ADSCs in a dose-dependant manner [71].

In vitro exposure of human ADSCs to increasing doses of tamoxifen, a selective oestrogen receptor modulator used in breast cancer treatment, resulted in apoptosis, inhibition of proliferation and differentiation in a dose- and time-dependent manner [72]. Interestingly, Liang et al. [73] reported no difference in differentiation potential of ADSCs in vitro when exposed to three commonly used chemotherapeutic agents: cisplatin, comptothecin and vincristine.

### Diabetes mellitus

A LOE-2 study compared gene expression profiles of ADSCs in diabetic patients to those in age- and BMI-matched controls. They reported a significant decrease in ADSC differentiation capacity and up-regulation of genes involved in inflammation and apoptosis in the diabetic patients [74]. Harris et al. [30] reported a trend of lower yields of ADSCs in diabetics (*n* = 18) which was not statistically significant.

Three LOE-5 animal studies investigated the effect of fat grafting in a diabetic setting. Choi et al. [75] demonstrated a higher resorption in rats with diabetes over 90 days. This was supported by Jung et al. [76] with reports of significantly lower weights, volumes and vascularity in the diabetic group compared to the control group. Ferrer-Lorente et al. [77] used a different approach and analysed gene expression in subcutaneous adipose tissue from Zucker diabetic fatty rats and their non-diabetic controls. The subcutaneous adipose tissue of diabetic rats displayed widespread downregulation of markers of stemness and differentiation and angiogenic potential.

## Discussion

This review aims to identify patient characteristics that may influence adipocyte and ADSC viability and behaviour in order to have a greater understanding of how to improve fat graft retention rates. Peer first postulated the ‘Cell Survival’ theory, suggesting the number of viable cells within the graft correlated with the long-term survival of grafts [78, 79]. The restorative and reconstructive qualities of fat grafting have been attributed to ADSCs within the graft [80]. Moreover, addition of ADSCs to transplanted fat was reported to support the formation of new vasculature and promote graft retention [20, 41, 81].

A total of 41 papers were included in this review. Most were in vitro studies on human tissue (LOE-2) and used similar in vitro techniques to analyse the effects of different patient factors on adipocyte and ADSC count as well as function. However, it is difficult to make conclusive recommendations as it is not clear whether the in vitro findings translate to clinically significant differences. Methods of adipocyte isolation and processing protocols also varied among the studies and this is known to affect yield [82, 83]. The included studies mostly had modest sample sizes and consisted of healthy patients undergoing elective plastic surgery procedures; therefore, the homogeneity in the sample may have reduced the power. Most studies did not report on other ADSC functions such as immunodulatory or angiogenic properties.

With an aging population, fat transfer procedures, particularly for regenerative properties, are becoming more relevant. Age-related changes in fat tissue inflammatory profiles resemble those in obesity, in which senescent stem cells and endothelial cells accumulate along with an increase in circulating pro-inflammatory cytokines, including TNFα and IL-6 [84, 85]. This increased cytokine release by ADSCs activates adjacent cells into a pro-inflammatory state, impeding adipogenesis and promoting fat cell lipolysis [86].

Advanced age is known to have detrimental effects on blood and BM-MSCs [87–89]. In contrast, ADSC yield seemed to be stable across age groups in 12 of 16 LOE-2 studies included in this review (Table 1). The subjects in most of these studies were having elective cosmetic procedures and therefore the homogeneity of subjects, with very few subjects being elderly (>70 years), may have reduced the power to detect an effect. However, it is reassuring to know that ADSC yield appears relatively stable across age groups. Similarly, BMI was also found to have little effect on ADSC yield in 12 LOE-2 studies. Although the absolute yield of precursor cells per gram of adipose tissue was reduced in some studies, this can be explained by the initial increase in adipocyte size seen with weight gain [90, 91]. These findings demonstrate the reproducibility of adipose tissue as a consistent and abundant source of ADSCs across a spectrum of ages and BMI values.

Unsurprisingly there is evidence to support reduced proliferative and differentiation capacities with increasing age [30, 34, 37, 38], which is likely related to the decreased susceptibility of precursor cells to respond to extracellular signals. Similarly, increasing BMI, particularly within the obese category (BMI >30 kg/m2), was observed to negatively impact ADSC functional capacities, with implications for their use in cellular therapies and reconstructive surgery (Table 2). Although larger in size, these adipocytes in obese individuals have been shown to be deficient in perilipin phosphoproteins, which are found on the cell surface and act as gatekeepers preventing lipases from hydrolyzing triacylglycerol [92]. This deficiency may contribute to fragile cell membranes, thereby potentially increasing the basal rate of lipolysis [93]. Another important consequence of adipocyte enlargement is the development of local inflammation with infiltration of monocytes/macrophages that act as scavengers of the remaining debris and lipids [94] and higher expression of pro-inflammatory proteins, including TNF-α, IL-6 and factor VII, affecting adipocyte survival and functional capacities in recipient sites [51, 95]. So far no clinical studies have investigated if these cellular changes translate into clinical differences. Graft enrichment through supplementation of ADSCs may be relevant for this cohort, along with weight reduction interventions.

No clinical studies have yet set out to address associations between gender, menopausal status and hormone replacement therapies and ADSC yield. Circulating oestrogen is a major regulator of adipose tissue, exerting its effects primarily through two oestrogen receptors (ERs), ER-α and ER-β. Studies have shown variable distribution of these receptors among fat depots, affecting responses to oestrogen signalling [96, 97]. Depletion of oestrogen levels, for instance in post-menopausal women or in ovariectomised mice, have been associated with an increase in lipolytic activity, adipocyte diameter, oxidative stress and inflammation [98].

In a questionnaire study among 508 surgeons practising in the US, the most preferred site for fat harvest was the abdomen (89%), followed by thighs (34%) [99]. So far, clinical studies have not yet been able to identify an ideal donor site. Therefore, when used as fillers, site choice may be made on ease, safety of access, fat abundance and patient preference. Nevertheless, application of grafts harvested from a single depot has been advised, especially for treatment of mirror zones (e.g. nasolabial folds, cheeks ) as there is in vitro evidence that adipocytes from different anatomical depots exhibit different morphology and functional capacity [33, 100]. For example, the distributions of adrenergic receptor subtypes on adipocytes vary between depots, with abdominal subcutaneous depots showing higher concentrations of adrenergic receptors than gluteo-femoral adipose tissue [101]. Therefore, abdominal depots in general are characterized by a higher lipid turnover and undergo increased lipolysis in response to adrenergic stress stimuli.

In comparison, lower-body fat stores have reduced lipid turnover, retain the capacity to recruit additional adipocytes as a result of weight gain, demonstrate fewer signs of inflammatory insult and tend to be more resistant to TNFα-induced apoptosis than abdominal ADSCs in in vitro studies [100, 102, 103]. New data suggest that these profound functional differences between upper-body and lower-body tissues are controlled by site-specific expression of developmental genes that direct both the degree of adipocyte proliferation and aspects of differentiation [65, 100, 104]. It is accepted, therefore, that adipocytes from different subcutaneous depots (abdominal versus gluteal) are developmentally distinct and are cell autonomous, which means that even after transplantation during fat transfer procedures, they can be expected to have distinct phenotypes [105, 106]. How they interact with the microenvironment in recipient sites has not yet been studied in detail. It is possible gene regulation determines depot-specific properties during development and sex steroids play a modulatory role [27].

Additional data are required to determine whether these findings translate into long-term retention. Most studies have rarely considered interactions between gender, BMI, menopausal status and other potential confounders and were of modest sample sizes (Table 3). Nevertheless, current clinical data suggest there is no significant difference in the volume or weight of grafted fat from different donor sites.

Radiotherapy is increasingly being used to treat numerous human malignancies [107]. Altered molecular signalling and formation of reactive oxygen species cause single-stranded DNA breaks that do not repair completely and activate premature senescence or accelerated terminal differentiation [108]. Despite improved resilience of ADSCs through their superior DNA damage repair mechanisms and reduced metabolic demands that protect them from hypoxia and subsequent apoptosis [109, 110], studies have demonstrated that radiotherapy adversely affects ADSCs, necessitating the introduction of non-irradiated progenitor cells from distant donor sites [70, 107].

Fat transfer in radiotherapy patients is further complicated by the fact that irradiated recipient sites have unfavourable microenvironments for graft survival because of hypoxia and chronic inflammatory states. Furthermore, stem cells within the injured area recruit myofibroblast-like cells, which in turn contribute to fibrosis [111]. The immunoregulatory capacity of transferred ADSCs to modulate inflammation and thereby reduce fibrosis and its normalising role in tissue regeneration have been well documented [112, 113].

Use of tamoxifen is routinely discontinued before and after major surgery because of the increased risk of venous thrombo-embolism with no documented effect on increased cancer risk [114]. Given that tamoxifen has a dose- and time-dependant detrimental effect on ADSCs, discontinuing its use to support fat engraftment and survival may be beneficial.

The link between chronic diseases like diabetes and impaired BM-MSC properties is well established [115–117]. Evidence collated from LOE-5 animal studies also supports the detrimental effects of diabetes on ADSC function, potentially limiting its potency in regenerative and reconstructive surgery [30, 74–77]. The effect of other common diseases and medications should also be further investigated. Evidence so far suggests no significant impact of total cholesterol, hypertension, renal disease, physical exercise and peripheral vascular disease on ADSC yield [30, 42].

Currently, surgeons aim to improve fat graft resorption by optimising the fat harvest and injection techniques and preparation of the recipient bed [118]. An improved understanding of patient factors that affect fat viability and function would assist in the identification of patient groups that could potentially benefit from graft enrichment techniques. Surgeons may consider transferring larger volumes of processed fat and adopt techniques to boost levels of ADSCs in grafts using techniques such as cell assisted lipotransfer [31, 37, 38], where part of the lipoaspirate is used to extract ADSCs, which are then used to supplement the cellular suspension before transplant.

Alternative strategies such as banking younger adipose tissue when biological activity is at its greatest potential or before chemoradiotherapy has become an option with cryopreservation techniques [119, 120]. Transfer of smaller fat particles and serial transfers of smaller volumes at closer intervals may also support adipocyte survival within adverse microenvironments by reducing demand [112]. The search for the ideal fat particle size for transfer is ongoing [79].

## Conclusions

Overall the literature is sparse, with varied methodologies used to compare the effects of different factors on adipocyte and ADSC functionality. A more uniform comparison of all factors highlighted in this review, with the application of a combination of tests for each outcome measure, is essential to fully understand factors that affect adipocyte and ADSC viability as well as function. This would be crucial information for surgeons when deciding appropriate volumes of lipoaspirate to inject, improve patient selection, and counsel patient expectations with regards to outcomes and likelihood for repeat procedures.

## Additional files

Additional file 1: Table S1. Search strategy summary. (DOC 32 kb)

## Abbreviations

ADSC: Adipose-derived stem cell
BM: Bone marrow
BMI: Body mass index
LOE: Level of Evidence
MSC: Mesenchymal stem cell
SVF: Stromal vascular fraction
