# Supplementary material for: Systematic review of patient factors affecting adipose stem cell viability and function: implications for regenerative therapy
Source: Stem Cell Res Ther. 2017 Feb 28;8:45. doi: 10.1186/s13287-017-0483-8 (PMC5329955; doi:10.1186/s13287-017-0483-8)
Supplement: Additional file 1: Table S1. — Search strategy summary. (DOC 32 kb) [file 13287_2017_483_MOESM1_ESM.doc]

**Table S**1: Search Strategy Summary

| **EMBASE SEARCH STRATEGY** |
| --- |
| Database: Embase Classic+Embase <1947 to 2016 February 09>  Search Strategy:  --------------------------------------------------------------------------------  1 (adipos* or adipocyte* or preadipocyte*).tw. (126449)  2 fat transfer.tw. (297)  3 exp Adipocytes/ (37246)  4 exp Adipose Tissue/ (131766)  5 Adipogenesis/ (8332)  6 or/1-5 (198420)  7 exp stem cell/ (248843)  8 ((stem or mesenchymal) adj2 cell*).tw. (279698)  9 exp mesenchymal stroma cell/ (6789)  10 or/7-9 (358858)  11 exp Adipocytes/tr or exp Adipose Tissue/tr (0)  12 (6 and 10) or 11 (16641)  13 *Adipogenesis/ or exp *Adipose Tissue/ or exp *Adipocytes/ or (adipos* or adipocyte* or preadipocyte* or fat transfer).ti. (73886)  14 10 and 13 [ focussed adipose search + stem cells] (7793)  15 viability.ti. or exp *cell viability/ or exp *tissue graft/ or exp *tissue transplantation/ or *cell survival/ (242110)  16 exp Aging/ or aging.tw. (285547)  17 (bmi or body mass index* or obesity or obese).tw. (474786)  18 exp obesity/ or body mass/ (511968)  19 (estrogen* or oestrogen*).tw. or exp Estrogen/ or exp Menopause/ (360612)  20 radiotherap*.tw. or exp radiotherapy/ (545482)  21 (smok* or nicotine*).tw. or exp smoking/ (382062)  22 exp Diabetes Mellitus/ or diabet*.tw. (851311)  23 donor site/ or donor site$1.tw. (14651)  24 or/16-23 [ various aspects ] (2688242)  25 24 and 12 [ various aspects + ADSC ] (3025)  26 15 and 14 [ focussed adipose search + stem cells + tissue viability ] (896)  27 25 or 26 [ combined search ] (3813)  28 27 and 15 [ limiting all to tissue viability ] (1038) |
| **OVID SEARCH STRATEGY** |
| Database: Ovid MEDLINE(R) In-Process & Other Non-Indexed Citations and Ovid MEDLINE(R) <1946 to Present>  Search Strategy:  --------------------------------------------------------------------------------  1 (adipos* or adipocyte* or preadipocyte*).tw. (92096)  2 fat transfer.tw. (269)  3 exp Adipocytes/ (16463)  4 exp Adipose Tissue/ (77671)  5 Adipogenesis/ (3932)  6 or/1-5 (131671)  7 exp stem cells/ (154625)  8 ((stem or mesenchymal) adj2 cell*).tw. (190341)  9 Mesenchymal Stromal Cells/ (20339)  10 or/7-9 (254373)  11 exp Adipocytes/tr or exp Adipose Tissue/tr (3043)  12 (6 and 10) or 11 (13473)  13 *Adipogenesis/ or exp *Adipose Tissue/ or exp *Adipocytes/ or (adipos* or adipocyte* or preadipocyte* or fat transfer).ti. (62417)  14 10 and 13 [ focussed adipose search + stem cells] (6053)  15 viability.tw. or Tissue Survival/ or cell survival/ or Graft Survival/ (270555)  16 exp Aging/ or aging.tw. (275464)  17 (bmi or body mass index* or obesity or obese).tw. (301388)  18 obesity/ or obesity, abdominal/ or obesity, morbid/ or pediatric obesity/ or body mass index/ (208382)  19 (estrogen* or oestrogen*).tw. or Estrogens/ or Menopause/ (170763)  20 radiotherap*.tw. or exp radiotherapy/ or exp Stem Cells/re [Radiation Effects] (228312)  21 (smok* or nicotine*).tw. or exp smoking/ (261642)  22 exp Diabetes Mellitus/ or diabet*.tw. (515163)  23 or/16-22 [ various aspects ] (1620199)  24 23 and 12 [ various aspects + ADSC ] (1822)  25 15 and 14 [ focussed adipose search + stem cells + tissue viability ] (686)  26 24 or 25 [ combined search ] (2437)  27 26 and 15 [ limiting all to tissue viability ] (732)  *************************** |
